# Influence of the autotaxin-lysophosphatidic acid axis on cellular function and cytokine expression in different breast cancer cell lines

**DOI:** 10.1038/s41598-022-09565-3

**Published:** 2022-04-01

**Authors:** Theresa Hauck, Sheetal Kadam, Katharina Heinz, Maria Garcia Peraza, Rafael Schmid, Andreas E. Kremer, Katharina Wolf, Alina Bauer, Raymund E. Horch, Andreas Arkudas, Annika Kengelbach-Weigand

**Affiliations:** 1 Department of Plastic and Hand Surgery, University Hospital Erlangen, Friedrich-Alexander-Universität Erlangen-Nürnberg, Krankenhausstr. 12, 91054 Erlangen, Germany; 2 Department of Medicine 1, University Hospital Erlangen, Friedrich-Alexander-Universität Erlangen-Nürnberg, Ulmenweg 18, 91054 Erlangen, Germany; 3 Department of Gastroenterology and Hepatology, University Hospital Zürich, Rämistrasse 100, 8091 Zürich, Switzerland

**Keywords:** Cancer, Medical research, Molecular medicine

## Abstract

Previous studies provide high evidence that autotaxin (ATX)-lysophosphatidic acid (LPA) signaling through LPA receptors (LPAR) plays an important role in breast cancer initiation, progression, and invasion. However, its specific role in different breast cancer cell lines remains to be fully elucidated to offer improvements in targeted therapies. Within this study, we analyzed in vitro the effect of LPA 18:1 and the LPAR1, LPAR3 (and LPAR2) inhibitor Ki16425 on cellular functions of different human breast cancer cell lines (MDA-MB-231, MDA-MB-468, MCF-7, BT-474, SKBR-3) and the human breast epithelial cell line MCF-10A, as well as Interleukin 8 (IL-8), Interleukin 6 (IL-6) and tumor necrosis factor (TNF)-alpha cytokine secretion after LPA-incubation. ATX-LPA signaling showed a dose-dependent stimulatory effect especially on cellular functions of triple-negative and luminal A breast cancer cell lines. Ki16425 inhibited the LPA-induced stimulation of triple-negative breast cancer and luminal A cell lines in variable intensity depending on the functional assay, indicating the interplay of different LPAR in those assays. IL-8, IL-6 and TNF-alpha secretion was induced by LPA in MDA-MB-468 cells. This study provides further evidence about the role of the ATX-LPA axis in different breast cancer cell lines and might contribute to identify subtypes suitable for a future targeted therapy of the ATX-LPA axis.

## Introduction

Autotaxin (ATX), also referred as ectonucleotide pyrophosphatase/phosphodiesterase 2 (ENPP2), is a secreted plasma lysophospholipase D which produces lysophosphatidic acid (LPA) by hydrolyzing lysophosphatidylcholine (LPC)^1,2^. It is considered the major source of synthetized LPA and reacts locally rather than systemically^3^. The produced LPA is a bioactive phospholipid and signals through its interaction with at least six specific G protein-coupled LPA receptors (LPAR1–6) (reviewed e.g. in^4^). Besides physiological properties, e.g. during vascular and embryonic development, LPA plays a decisive role in malignant tumor diseases^5^. Enhanced expression of ATX and upregulation of LPAR have been reported in various types of cancer, including breast cancer, ovarian cancer, glioblastomas, prostate cancer, etc.^6^. Breast cancer is a very heterogeneous type of cancer, which varies widely in cancer development, progression, and resistance to chemotherapeutics or radiotherapy among patients. Due to this heterogeneity, additional targeted therapeutic approaches are required. The ATX-LPA signaling axis might thereby play an important role. E.g. previous studies found that ATX and LPA promote resistance to the effectiveness of taxol^7,8^. Further, it was revealed that ATX-LPA signaling could protect breast cancer cells against radiotherapy by stimulating a wound healing response (reviewed in^9,10^).

Previous in vitro and in vivo studies provided high evidence that ATX-LPA signaling through LPAR plays an important role in breast cancer. Especially LPAR1–3 were identified in a variety of breast cancer cell lines to promote proliferation, migration, and invasion and might participate in the initiation of breast cancer^11,12^. In particular, LPAR1 and LPAR2 are described to be expressed in benign and malignant breast cancer tissues^13^. Further, previous data revealed that the expression of LPAR3 is related to enhanced metastatic ability and is increased mainly in triple-negative breast cancers (TNBC)^14,15^. Ki16425 is a competitive inhibitor for LPAR1, LPAR3, and with a low potency for LPAR2^16^. It was found to inhibit migration, invasion, and transmigration of TNBC cells in vitro and to suppress bone metastases in in vivo breast cancer models^14,17^.

Although ATX levels are elevated in tumor tissue, breast cancer cells themselves are poor producers of ATX compared to surrounding tumor-associated fibroblasts or adipose tissue^10,18,19^. The secreted ATX stimulates breast cancer cells via different LPAR, whereupon the breast cancer cells produce cytokines in increasing amounts. In a vicious circle, these cytokines, in turn, stimulate the adjacent adipose tissue to produce more ATX^19–21^. Intervening in this vicious circle by antagonizing LPAR or inhibiting ATX represents a promising approach in targeted tumor therapy. However, the role of ATX-LPA signaling and the secretion of cytokines particularly with respect to different breast cancer subtypes remains to be fully elucidated to offer improvements in targeted therapies. Therefore, the aim of this study was to analyze the influence of the ATX-LPA axis on the functional properties and cytokine secretion of different mammary carcinoma cell lines.

## Materials and methods

### Breast cancer cell lines and cell culture

Five different breast cancer cell lines from American Type Culture Collection, ATCC (Manassas, VA, USA) were used. The following cell lines and media were used: MDA-MB-231 (ATCC HTB-26, TNBC, mesenchymal-like) and MCF-7 (ATCC HTB-22, luminal A): DMEM (Gibco, Life Technologies, Carlsbad, CA, USA), 10% fetal bovine serum (FBS), 1% l-glutamine, and 1% non-essential aminoacids; MDA-MB-468 (ATCC HTB-132, TNBC, basal-like): DMEM/F12 (Biochrom AG, Berlin, Germany), 10% FBS; BT-474 (ATCC HTB-20, luminal B): Hybri-Care Medium (ATCC), 10% FBS, 1.5 g/l NaHCO_3_ (Sigma Aldrich, St. Louis, MO, USA); SKBR-3 (ATCC HTB-30, HER2-positive): McCoy’s 5a Modified Medium (Thermo Fisher Scientific Inc., Waltham, MA, USA), 10% FBS. MCF-10A (ATCC CRL-10317) were used as healthy mammary epithelial cell line cultivated in Mammary Epithelial Cell Growth Medium (PromoCell GmbH, Heidelberg, Germany) with the addition of 100 ng/ml cholera toxin (Sigma Aldrich), 5 µg/ml insulin, 0.5 µg/ml hydrocortisone, 10 ng/ml epidermal growth factor (EGF), and 0.004 ml/ml bovine pituitary extract (BPE). All mediums contained 1% penicillin/streptomycin. All cells were cultivated at 37 °C and 5% CO_2_. Medium was changed every 2–3 days.

### Experimental groups

For measuring the effect of Oleoyl-LPA (LPA 18:1, 857130P; Avanti Polar Lipids Inc., Alabaster, AL, USA) and the LPA receptor 1, 3 (and 2) blocker Ki16425 (Sigma Aldrich) on cell properties, different experimental groups were performed summarized in Table 1. LPA was dissolved according to manufacturer’s instructions. Cells were seeded in the corresponding medium supplemented with either 0.1 μM LPA 18:1 or 1 μM LPA 18:1. 2 μM or 20 μM Ki16425 was added one minute before treatment with LPA. For all experimental groups including the control group and in all assays 10% FBS in culture medium was replaced by 0.2% fatty acid free bovine serum albumin (BSA; Sigma Aldrich).

**Table 1:** Experimental groups.

Group	LPA 18:1	Ki16425
1 (control)	–	–
2	–	2 µM
3	–	20 µM
4	0.1 µM	–
5	1 µM	–
6	0.1 µM	2 µM
7	1 µM	2 µM
8	0.1 µM	20 µM
9	1 µM	20 µM

### Proliferation assay

MDA-MB-231 cells were seeded at a density of 1 × 10^3^ cells/well, MDA-MB-468, MCF-7, BT-474, SKBR-3 and MCF-10A were seeded at a density of 5 × 10^3^ cells/well in 96-well plates in triplicate. Every 24 h, the medium was replaced according to the experimental groups, thus, including fresh LPA (Table 1). After an incubation period of 24 h, 48 h, and 72 h, cell proliferation was measured by fluorometric quantification of DNA using CyQUANT Direct Cell Proliferation Assay Kit (Invitrogen/Thermo Fisher, Carlsbad, CA, USA) according to the manufacturer’s instructions. 100 μl of 2X detection reagent was added to each well. After the incubation period of 60 min at 37 °C and 5% CO_2_, fluorescence was measured at 480/535 nm (NOVOstar, BMG LABTECH, Ortenberg, Germany). The assay was performed three times for all cell lines.

### Migration

For analysis of cell migration, the Oris Assembly Kit (Platypus Technologies, Madison, WI, USA) was used. Cell seeding stoppers were inserted into the wells of a 96-well plate for creating a cell-free area. 5 × 10^4^ MDA-MB-231, 7.5 × 10^4^ MDA-MB-468, 7.5 × 10^4^ MCF-7, 1 × 10^5^ SKBR-3, 1 × 10^5^ BT-474, or 1 × 10^5^ MCF-10A were seeded in technical triplicate in their standard cell culture medium and incubated for 24 h at 37 °C and 5% CO_2_. After 24 h, the stoppers were removed carefully. The medium was removed, the cells were washed twice with PBS and medium was changed accordingly to the experimental groups (Table 1). Every 24 h, the medium was replaced according to the experimental groups, thus, including fresh LPA. Images in the central region were taken in 40-fold magnification at 0 h, 24 h, 48 h, and 72 h (Olympus IX83, cellSens Software, Olympus Corporation, Tokio, Japan). Migrated cells were semi-automatically measured using Fiji Is Just ImageJ (Fiji)^22^. For quantification, control at 24 h was set 1. The assay was performed three times for all cell lines.

### Invasion and transmigration assay

For invasion and transmigration assays, transwell inserts with a pore size of 8 μm were used (ThinCert, Greiner Bio-One GmbH, Frickenhausen, Germany). The transwells for invasion assays were coated with 2.4 mg/ml collagen type I from bovine skin (Sigma-Aldrich). The transwells were placed into a 24-well plate and seeded in technical triplicate with 1 × 10^5^ cells in 300 μl of the corresponding control medium. The lower chamber was filled with medium according to the experimental groups (Table 1). After 8 h at 37 °C and 5% CO_2_, the transwell inserts were washed with PBS, fixed with ice-cold methanol and stained with 1 μg/ml 4′,6-diamidino-2-phenylindole (DAPI) for 10 min (Life technologies, Carlsbad, CA, USA). Non-migrated or non-invaded cells in the inner part of the transwells were removed by a PBS-coated cotton swab wiped in twisting motions. Transmigrated and invaded cells were counted in the four quadrants of each transwell in 40-fold magnification (Olympus IX83, cellSens Software). The assay was performed two times for all cell lines (control was set 1).

To investigate the influence of the LPA concentration on the transmigration rate of MDA-MB-231 cells, a seven point dose response curve was performed. Therefore, transmigration assay was conducted as described above, whereby 0 µM (control), 0.05 µM, 0.1 µM, 0.5 µM, 1 µM, 5 µM, and 10 µM LPA were added to the control medium in the lower chamber. This assay was performed in technical triplicate and in two replicate experiments.

### Real-time qPCR

For analysis of LPAR1–3 expression of different cell lines, RNA was extracted with the RNeasy Mini Kit (Qiagen, Hilden, Germany) and reverse transcribed into cDNA by using the QuantiTect Reverse Transcription Kit with a DNase I incubation (Qiagen). Quantitative real-time PCR was performed with the SsoAdvanced Universal SYBR Green Supermix (Bio-Rad Laboratories, Hercules, CA, USA) in a Light Cycler (Bio-Rad CFX96). All kits were used according to the manufacturers’ recommendation. Detected transcript levels were normalized to the four different housekeeping genes Coiled-Coil Serine Rich Protein 2 (CCSER2), Symplekin Scaffold Protein (SYMPK), Ankyrin Repeat Domain 17 (ANKRD17), and Pumilio RNA Binding Family Member 1 (PUM1) using the 2^−ΔCT^-method. Primers were selected according to Tilli et al.^23^ and are summarized in Table 2. The assay was performed in technical triplicate and in three replicate experiments.

**Table 2:** Primer sequences.

Gene	Forward	Reverse
*LPAR1*	TTTATGAAGCTCCCCATCCACC	TGAACACGCCCCAGAACTAC
*LPAR2*	TACCGAGAGACCACGCTCAG	GCCTAAACCATCCAGGAGCA
*LPAR3*	GGTGAACGTGAGCGGATGT	AGCTTTGTTCCTGTCCAGTCA
*CCSER2*	GACAGGAGCATTACCACCTCAG	CTTCTGAGCCTGGAAAAAGGGC
*SYMPK*	CTTCACCAAGGTTGTGCTGGAG	GCGCTTGAAGATCAGGTCTCGA
*ANKRD17*	CAAATGGTGGACACCTCGATGTG	CTAAGTAGCGCACCACCTTCAC
*PUM1*	CTTTGGCAGAACGGATTCGAG	TCTCATTCTGCTGGTCTGAAGG

### Enzyme-linked immunosorbent assay (ELISA) measurements

6 × 10^5^ cells (MDA-MB-468, SKBR-3, BT-474, MCF-10A) or 5 × 10^5^ cells (MDA-MB-231, MCF-7), respectively, were seeded in T25 cell culture flasks in their standard culture medium. When reaching 80% confluency, the different breast cancer cell lines were stimulated with 1.0 μM LPA in culture medium containing 0.2% fatty acid free BSA. After 24 h, supernatants from all cell lines were collected and levels of secreted IL-8 were detected by highly sensitive IL-8 ELISA Kit (IL-8 Human ELISA Kit; Invitrogen, Waltham, MA, USA) according to the manufacturer’s instructions using the NOVOstar. Levels of secreted IL-6 and TNF-alpha were detected by using IMMULITE 1000 Immunoassay System (Siemens, Germany). According to the standard instructions, 100 μl of cell culture supernatants for IL-6 and TNF-alpha and 50 μl of cell culture supernatants for IL-8 measurements was used. The assay was performed in three replicate experiments.

### Statistics

For cell functional assays, differences between groups were analyzed using the Kruskal–Wallis test, followed by Dunn’s test for post-hoc analysis (GraphPad Prism version 8.3.0 for Windows; La Jolla, CA, USA). ELISA assays were analyzed using the Mann–Whitney U test; the asymptotic significance was used (SPSS v.21.0 Software/IBM, Armonk, NY, USA). Error bars in the graphs indicate the standard deviations (SD). A p-value ≤ 0.05 was considered significant.

## Results

### LPA significantly stimulated proliferation of MDA-MB-231 and MDA-MB-468 cells

**Figure 1:**
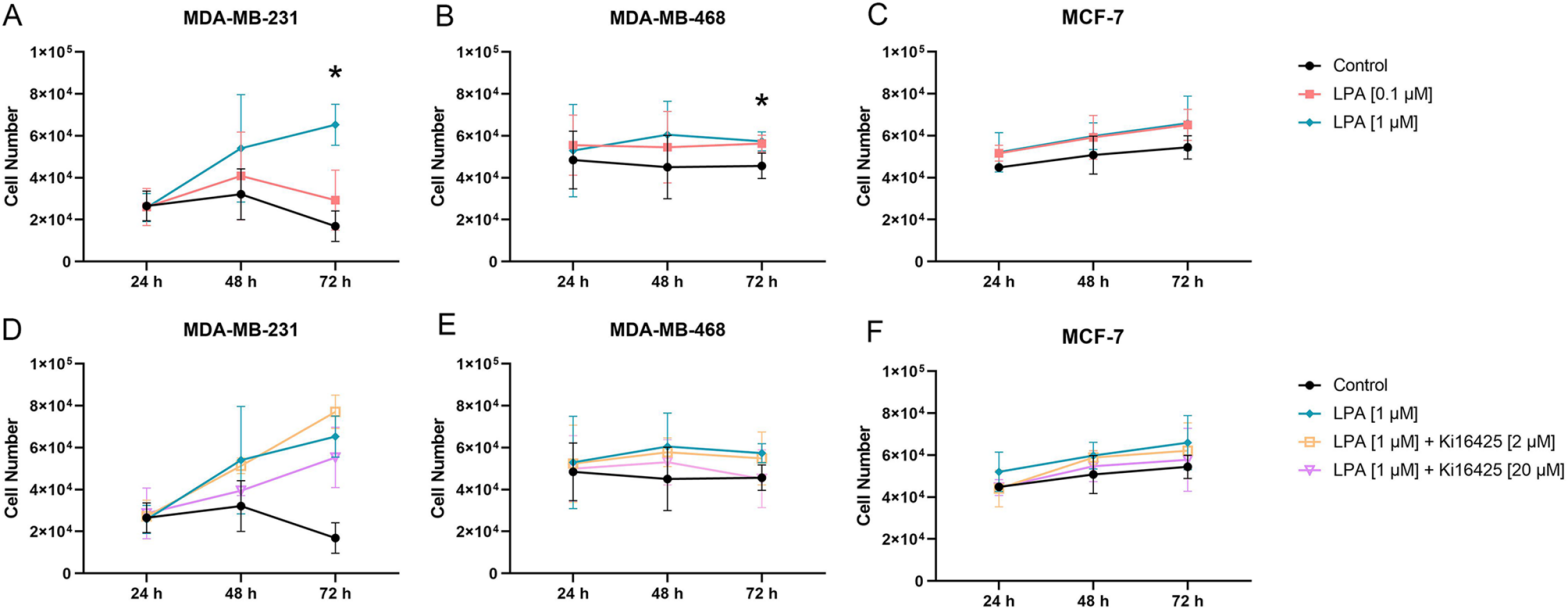
CyQUANT proliferation assay of breast cancer cells. Effect of lysophosphatidic acid (LPA) 18:1 and the LPA receptor 1, 3 (and 2) inhibitor Ki16425 on proliferation of MDA-MB-231, MDA-MB-468 and MCF-7 cell lines after 24 h, 48 h, and 72 h. **(A–C)** Data points show the absolute number of living cells per well (y-axis) with the addition of varying concentrations of LPA compared to the control group for several time points (x-axis). **(D–F)** Data points show the absolute number of live cells per well (y-axis) with the addition of 1.0 µM LPA and Ki16425 in varying concentrations as well as the control group for several time points (x-axis). n = 3 replicate experiments, values are presented as mean ± SD. *: p ≤ 0.05 compared to the control group (Kruskal–Wallis test and post-hoc Dunn’s test).

LPA induced significantly enhanced proliferation of MDA-MB-231 cells after 72 h. Proliferation of MDA-MB-468 cells was slightly but significantly stimulated by LPA after 72 h (Table 3, Fig. 1). High concentrated Ki16425 trended to inhibit this stimulatory effect (data of LPA 0.1 μM ± Ki16425 2 μM/20 μM not shown). Further, there was a stimulatory trend (= statistically non-significant) of LPA in MCF-7 cells. In contrary, LPA did not stimulate cell proliferation of BT-474, SKBR-3, and MCF-10A cells (Table 3). Ki16425 alone had no significant effect on cell proliferation (Supplementary Fig. S1).

**Table 3:** Cell proliferation. Effect of lysophosphatidic acid (LPA) 18:1 and the LPA receptor 1, 3 (and 2) inhibitor Ki16425 on cell proliferation after 24 h, 48 h and 72 h of different human breast cancer cell lines. LPA stimulated significantly proliferation of MDA-MB-231 and MDA-MB-468 cells, and trended to stimulate proliferation of MCF-7 cells.

Group	MDA-MB-231	MDA-MB-468	MCF-7	BT-474	SKBR-3	MCF-10A
LPA 0.1 μM	+	+	** + **	Ø	Ø	Ø
LPA 1.0 µM	++ p = 0.03 (72 h)	++ p = 0.05 (72 h)	** + **	Ø	Ø	Ø
LPA 0.1 μM + Ki16425 2 μM	Ø	Ø	Ø	Ø	Ø	Ø
LPA 1.0 μM + Ki16425 2 μM	Ø	Ø	Ø	Ø	Ø	–
LPA 0.1 μM + Ki16425 20 μM	Ø	Ø	Ø	Ø	Ø	Ø
LPA 1.0 μM + Ki16425 20 μM	–	–	–	Ø	Ø	–

n = 3 replicate experiments, a p-value ≤ 0.05 was considered significant (Kruskal–Wallis test and post-hoc Dunn’s test).

Ø: no effect; +: stimulatory trend; ++: statistically significant stimulation; –: inhibitory trend. For LPA 0.1 μM and LPA 1.0 μM, stimulation was compared to the control group; for LPA 0.1 μM/LPA 1.0 μM + Ki16425 2/20 μM inhibition was compared to the respective LPA group without Ki16425.

### LPA showed a stimulatory effect on the migration of MDA-MB-231, MCF-7, and SKBR-3 cells

**Figure 2:**
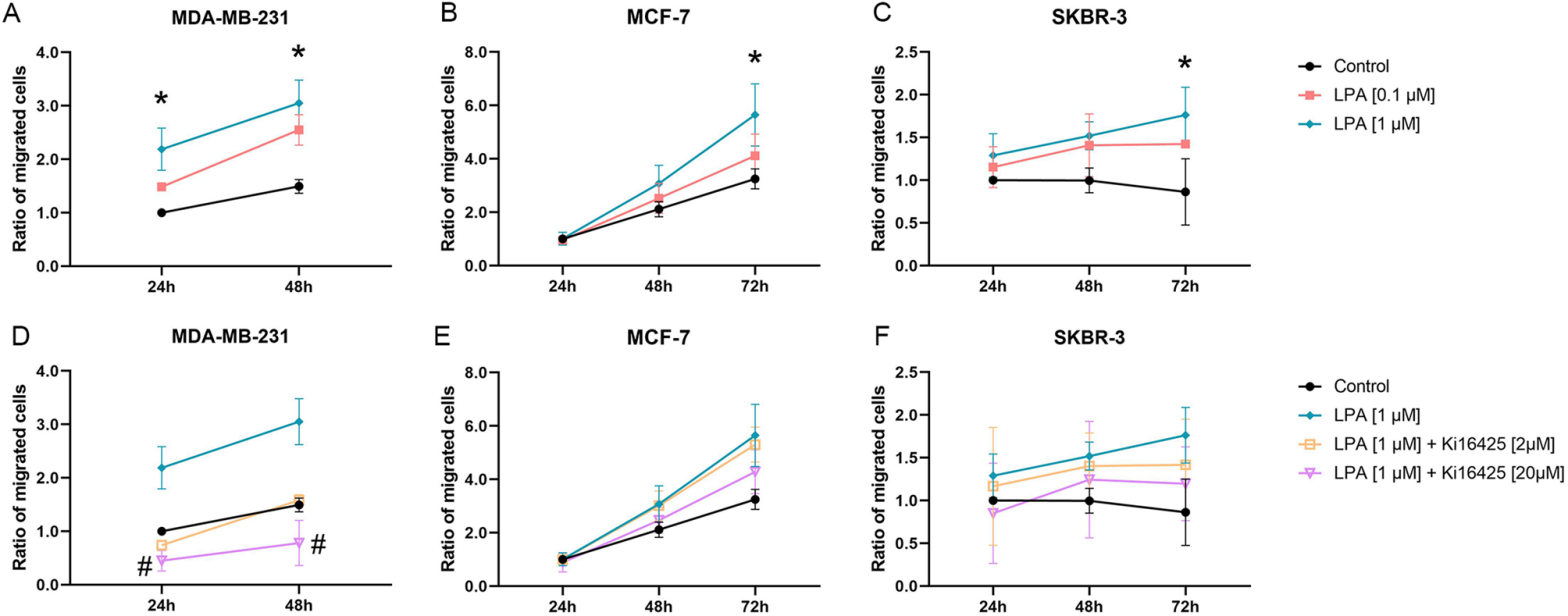
Migration assay. Effect of lysophosphatidic acid (LPA) 18:1 and the LPA receptor 1, 3 (and 2) inhibitor Ki16425 on migration after 24 h, 48 h, and 72 h of different human breast cancer cell lines (control 24 h = 1). **(A–C)** Data points show the ratio of migrated cells (y-axis) with the addition of varying concentrations of LPA compared to the control group for several time points (x-axis). **(D–F)** Data points show the ratio of migrated cells (y-axis) with the addition of 1.0 µM LPA and Ki16425 in varying concentrations as well as the control group for several time points (x-axis). n = 3 replicate experiments, values are presented as mean ± SD. *: p ≤ 0.05 compared to the control group; #: p ≤ 0.05 compared to 1.0 μM LPA (Kruskal–Wallis test and post-hoc Dunn’s test).

A significant stimulatory effect of LPA on migration was observed in MDA-MB-231, MCF-7, and SKBR-3 cells (Fig. 2, Table 4). High-concentrated Ki16425 inhibited these stimulatory effects significantly in MDA-MB-231 and slightly reduced stimulation in MCF-7 and SKBR-3 cells (data of LPA 0.1 μM ± Ki16425 2 μM/20 μM not shown). The sole addition of high-concentrated Ki16425 had no effect on migration rates of the cells (Supplementary Fig. S1). All time points of MDA-MB-468 and 72h time point of MDA-MB-231 were excluded from analysis since there was no clear migration front.

**Table 4:** Migration assay.

Group	MDA-MB-231	MCF-7	BT-474	SKBR-3	MCF-10A
LPA 0.1 μM	+	+	Ø	+	Ø
LPA 1.0 μM	++ p = 0.01 (24 h) p = 0.03 (48 h)	++ p = 0.03 (72 h)	Ø	++ p = 0.02 (72 h)	Ø
LPA 0.1 μM + Ki16425 2 μM	–	Ø	Ø	Ø	Ø
LPA 1.0 μM + Ki16425 2 μM	–	Ø	Ø	Ø	Ø
LPA 0.1 μM + Ki16425 20 μM	– – p = 0.01 (24 h) p = 0.02 (48 h)	–	Ø	–	Ø
LPA 1.0 μM + Ki16425 20 μM	– – p = 0.01 (24 h) p = 0.01 (48 h)	–	Ø	–	Ø

Effect of lysophosphatidic acid (LPA) 18:1 and the LPA receptor 1, 3 (and 2) inhibitor Ki16425 on migration after 24 h, 48 h, and 72 h of different human breast (cancer) cell lines. n = 3 replicate experiments, a p-value ≤ 0.05 was considered significant (Kruskal–Wallis test and post-hoc Dunn’s test).

Ø: no effect; +: stimulatory trend; ++: statistically significant stimulation; –: inhibitory trend; – –: statistically significant inhibition. For LPA 0.1 μM and LPA 1.0 μM stimulation was compared to the control group; for LPA 0.1 μM/LPA 1.0 μM + Ki16425 2/20 μM inhibition was compared to the respective LPA group without Ki16425.

### LPA led to slightly enhanced invasion of MDA-MB-231, MDA-MB-468, and MCF-7 cells

**Figure 3:**
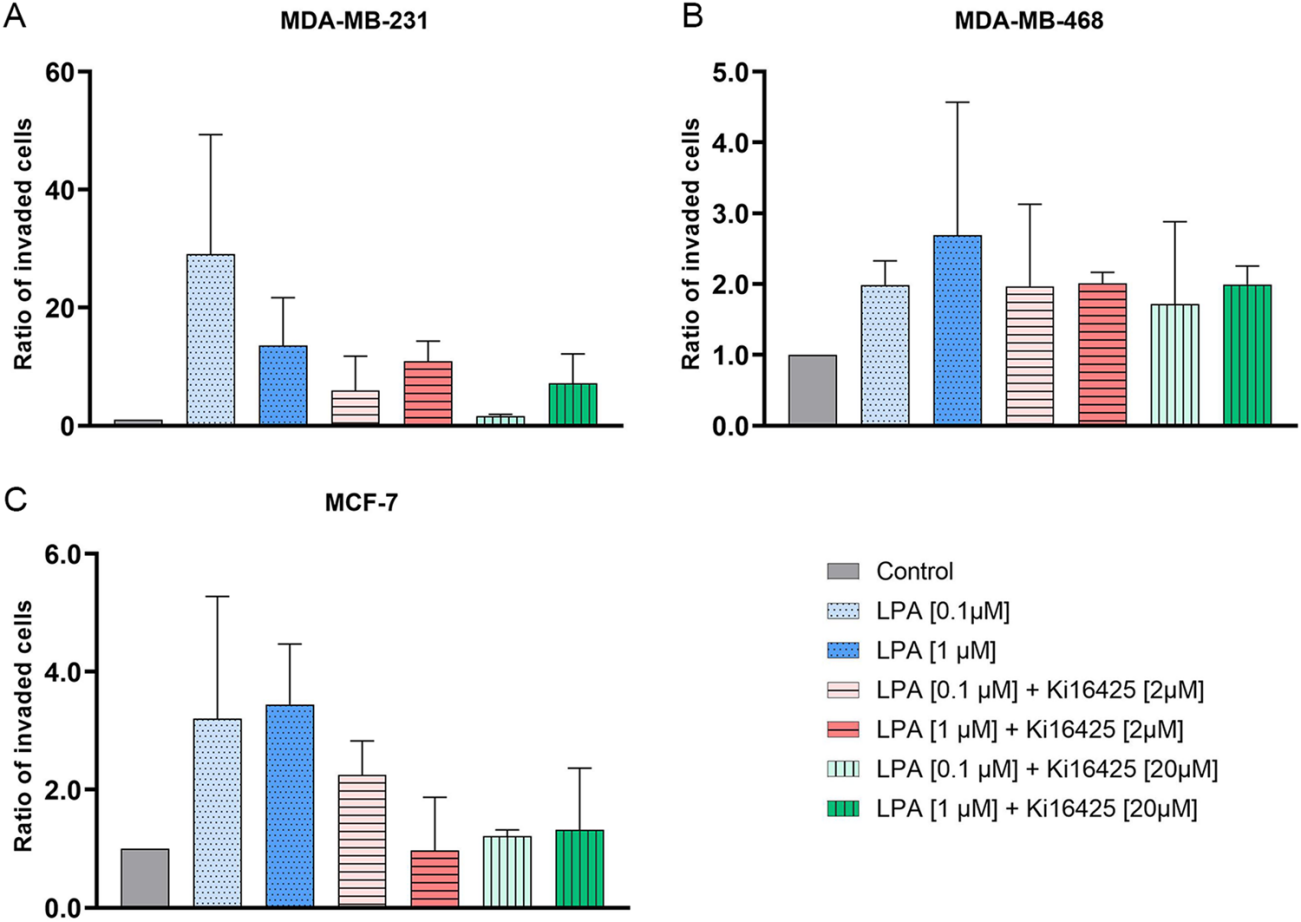
Invasion assay. Effect of lysophosphatidic acid (LPA) 18:1 and the LPA receptor 1, 3 (and 2) inhibitor Ki16425 on invasion of different human breast cancer cell lines (control = 1). **(A–C)** Columns show the ratio of invaded cells compared to the control group (y-axis) in varying concentrations of LPA and Ki16425 (x-axis). n = 2 replicate experiments, values are presented as mean ± SD. For LPA 0.1 μM and LPA 1.0 μM stimulation was compared to the control group; for LPA 0.1 μM/LPA 1.0 μM + Ki16425 2/20 μM inhibition was compared to the respective LPA group without Ki16425.

Invasion assays did not reveal any statistically significant differences between the experimental groups within the different cell lines (Fig. 3, Table 5). LPA trended to stimulate invasion of TNBC and MCF-7 cells, whereas invasion of SKBR-3, BT-474, and MCF-10A was not stimulated or even inhibited by LPA. In MDA-MB-231 cells, high-concentrated LPA could not be inhibited by Ki16425. However, Ki16425 had a slightly decreasing effect on LPA-induced invasion of MCF-7 cells. The stimulatory trend in MCF-7 cells indicates the involvement of LPAR2, and the stimulatory trend in MDA-MB-231 cells implies the involvement of LPAR1 in invasion. Ki16425 did not inhibit invasion of MDA-MB-468 (expression of mainly LPAR3 and LPAR2), which indicates a minor role of LPAR3 in invasion. The addition of Ki16425 alone had no effect on invasion of MDA-MB-231, BT-474, and MCF-10A, and a slightly stimulatory effect on MDA-MB-468, SKBR-3, and MCF-7 (Supplementary Fig. S1).

**Table 5:** Invasion assay.

Group	MDA-MB-231	MDA-MB-468	MCF-7	BT-474	SKBR-3	MCF-10A
LPA 0.1 μM	+	+	+	Ø	Ø	Ø
LPA 1.0 μM	+	+	+	–	Ø	–
LPA 0.1 μM + Ki16425 2 μM	**–**	Ø	Ø	Ø	Ø	Ø
LPA 1.0 μM + Ki16425 2 μM	Ø	–	–	Ø	Ø	Ø
LPA 0.1 μM + Ki16425 20 μM	**–**	Ø	**–**	Ø	Ø	Ø
LPA 1.0 μM + Ki16425 20 μM	Ø	–	–	Ø	Ø	Ø

Effect of lysophosphatidic acid (LPA) 18:1 and the LPA receptor 1, 3 (and 2) inhibitor Ki16425 on invasion of different human breast (cancer) cell lines. n = 2 replicate experiments.

Ø: no effect; +: stimulatory trend; –: inhibitory trend. For LPA 0.1 μM and LPA 1.0 μM stimulation was compared to the control group; for LPA 0.1 μM/LPA 1.0 μM + Ki16425 2/20 μM inhibition was compared to the respective LPA group without Ki16425.

### LPA induced particularly transmigration of MDA-MB-231 cells

**Figure 4:**
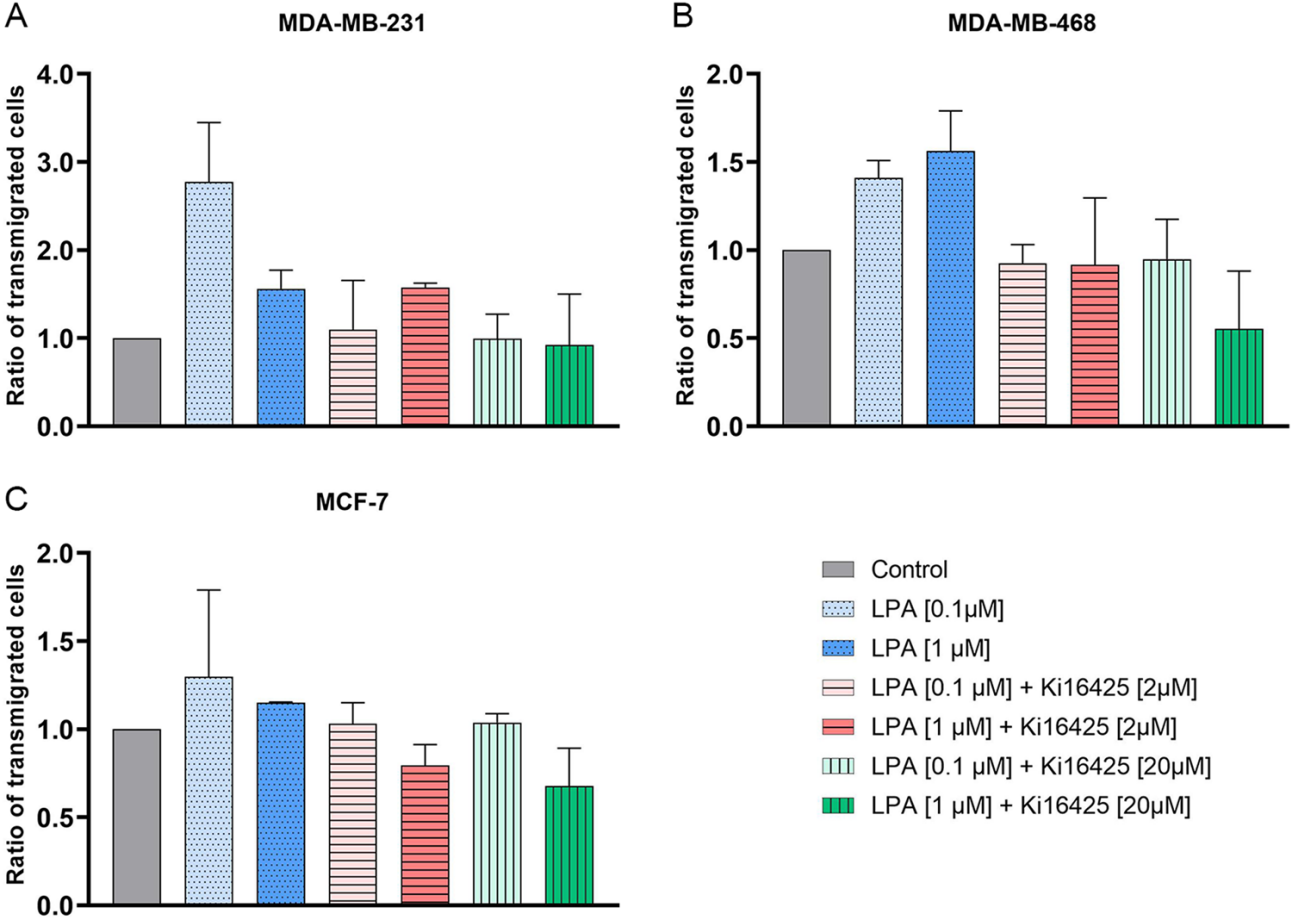
Transmigration assay. Effect of lysophosphatidic acid (LPA) 18:1 and the LPA receptor 1, 3 (and 2) inhibitor Ki16425 on transmigration of different human breast cancer cell lines (control = 1). **(A–C)** Columns show the ratio of transmigrated cells compared to the control group (y-axis) in varying concentrations of LPA and Ki16425 (x-axis). n = 2 replicate experiments. Values are presented as mean ± SD. For LPA 0.1 μM and LPA 1.0 μM stimulation was compared to the control group; for LPA 0.1 μM/LPA 1.0 μM + Ki16425 2/20 μM inhibition was compared to the respective LPA group without Ki16425.

There was a trend towards a stimulatory effect of LPA on transmigration of MDA-MB-231, MDA-MB-468 and MCF-7 cells (Fig. 4, Table 6). Ki16425 tended to inhibit this stimulatory effect of LPA. Transmigration of SKBR-3, BT-474 and MCF-10A were not stimulated by LPA. The addition of Ki16425 alone tended to stimulate transmigration of MDA-MB-231 and SKBR-3 cells and to inhibit transmigration of BT-474 cells (Supplementary Fig. S1). In MDA-MB-231 cells the higher concentration of LPA lead to a lower stimulatory effect compared to the lower concentration of LPA (Fig. 4). The transmigration rate of MDA-MB-231 in the dose response experiment increased up to 0.1 µM came to a plateau and decreased with higher concentrations (1.0–10.0 µM) of LPA (Supplementary Fig. S2).

**Table 6:** Transmigration assay.

Group	MDA-MB-231	MDA-MB-468	MCF-7	BT-474	SKBR-3	MCF-10A
LPA 0.1 μM	+	+	+	–	Ø	Ø
LPA 1.0 μM	+	+	+	–	Ø	Ø
LPA 0.1 μM + Ki16425 2 μM	**–**	–	–	Ø	Ø	Ø
LPA 1.0 μM + Ki16425 2 μM	Ø	–	–	Ø	Ø	Ø
LPA 0.1 μM + Ki16425 20 μM	**–**	–	–	Ø	Ø	Ø
LPA 1.0 μM + Ki16425 20 μM	–	–	–	Ø	Ø	Ø

Effect of lysophosphatidic acid (LPA) 18:1 and the LPA receptor 1, 3 (and 2) inhibitor Ki16425 on transmigration of different human breast (cancer) cell lines. n = 2 replicate experiments.

Ø: no effect; +: stimulatory trend; –: inhibitory trend. For LPA 0.1 μM and LPA 1.0 μM stimulation was compared to the control group; for LPA 0.1 μM/LPA 1.0 μM + Ki16425 2/20 μM inhibition was compared to the respective LPA group without Ki16425.

### MDA-MB-231 cells express high levels of LPAR1, MCF-7 cells express predominantly LPAR2, and LPAR3 expression on mRNA level was highest in MDA-MB-468 cells

**Figure 5:**
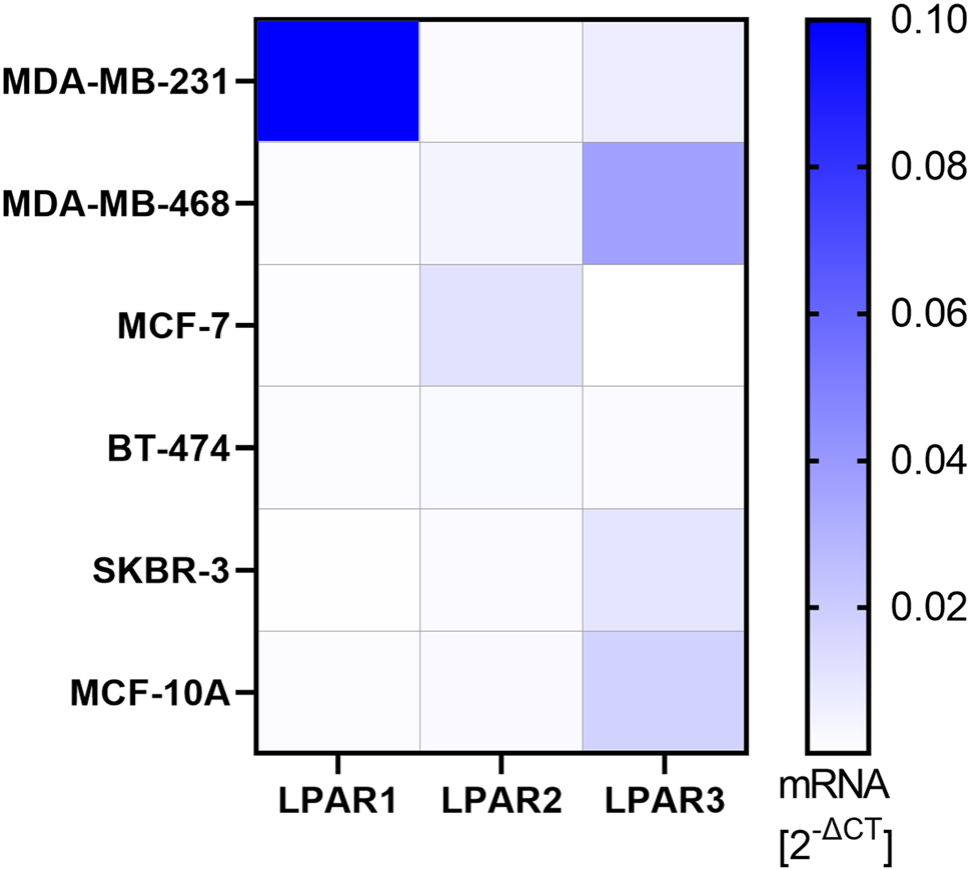
LPAR1–3 expression on mRNA level of different breast (cancer) cell lines. The heatmap shows relative LPAR mRNA expression compared to four different housekeeping genes (Coiled-Coil Serine Rich Protein 2 (CCSER2), Symplekin Scaffold Protein (SYMPK), Ankyrin Repeat Domain 17 (ANKRD17), and Pumilio RNA Binding Family Member 1 (PUM1)) using the 2^−ΔCT^-method. Values > 0.10 are presented in dark blue (relative LPAR1 mRNA expression of MDA-MB-231 is 0.60). n = 3 replicate experiments.

qPCR was performed to analyze the LPAR1–3 mRNA expression in different breast (cancer) cell lines (Fig. 5). LPAR1 was mainly expressed in MDA-MB-231 cells, whereas LPAR2 was particularly expressed in MCF-7 cells. LPAR3 was predominantly expressed in MDA-MB-468 cells. Expression levels of LPAR1–3 in BT-474, SKBR-3 and MCF-10A were low.

### LPA stimulated secretion of IL-8, IL-6 and TNF-alpha in MDA-MB-468 cells

**Figure 6:**
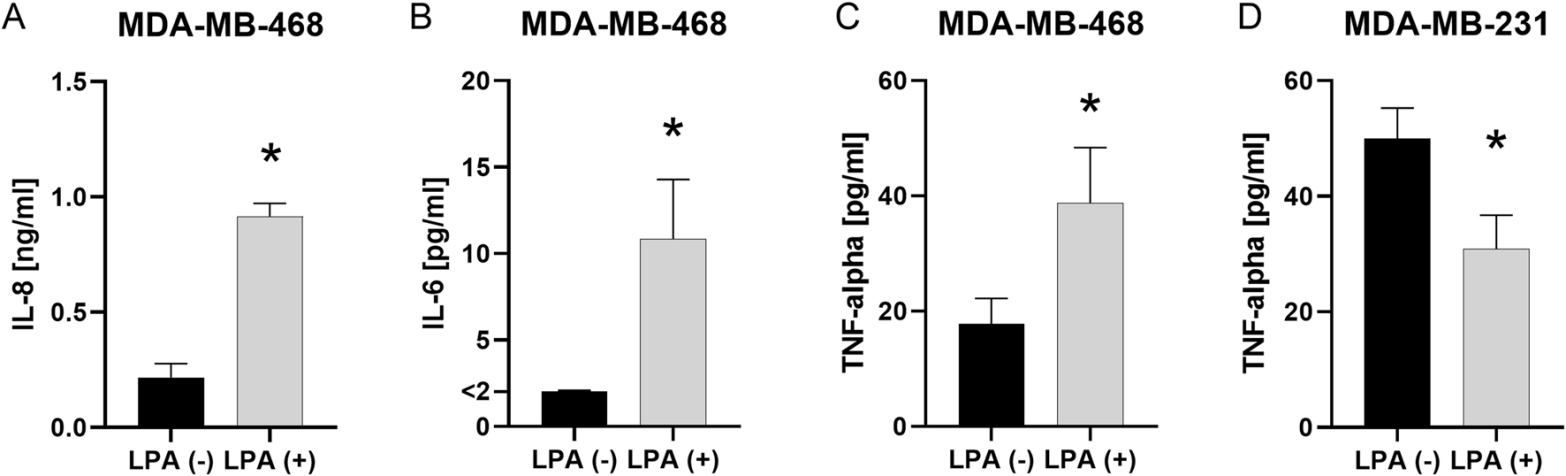
IL-8, IL-6 and TNF-alpha secretion after incubation with LPA. The secretion of IL-8, IL-6 and TNF-alpha of cell lines was measured with and without the addition of 1.0 μM lysophosphatidic acid (LPA) 18:1 using enzyme-linked immunosorbent assay (ELISA). **(A–D)** Columns show the concentration of **(A)** Interleukin 8 (IL-8), **(B)** Interleukin 6 (IL-6) and **(C, D)** tumor necrosis factor alpha (TNF-alpha) in the supernatant of **(A–C)** MDA-MB-468 and **(D)** MDA-MB-231 cells (y-axis) with and without the addition of LPA (x-axis). n = 3 replicate experiments. Values are presented as mean ± SD. *: p ≤ 0.05 (Mann–Whitney U test).

Compared to the non-stimulated controls, MDA-MB-468 showed enhanced secretion of IL-8 (p = 0.050), IL-6 (p = 0.046) and TNF-alpha (p = 0.050) after stimulation with LPA 1.0 μM (Fig. 6A–C; n = 3, Mann–Whitney U test). LPA did not stimulate the secretion of IL-8, IL-6 or TNF-alpha in any of the other cell lines. As LPAR2 is attributed an important role in the production and secretion of inflammatory mediators, insufficient expression of LPAR2 might have contributed to the lack of stimulation in other cell lines. However, in MDA-MB-231 cells, LPA 1.0 μM inhibited secretion of TNF-alpha (Fig. 6D, n = 3; p = 0.050).

## Discussion

The role of ATX-LPA signaling in malignancies and its involvement in tumor progression has been repeatedly shown in multiple studies so that the ATX-LPA axis evolved to a potential target of tumor therapies^24–26^. Breast cancer with its numerous subtypes represents one of the potential examples of targeted therapy. Despite extensive evidence that LPA is associated with tumorigenesis of breast cancer, a dedicated subtype analysis including different breast cancer cell lines and functional assays has not yet been performed. Previous studies have mostly examined the influence of the ATX-LPA axis on single cell functions of one or two subtypes. Thereby, various experimental designs lead to partially contradicting results^27,28^. Different experimental setups, including e. g. different incubation times, materials, and LPA concentrations, could lead to various results and impede the comparison of different cell lines among different studies. Therefore, the aim of the study was to elucidate the role of ATX-LPA signaling in different breast cancer subtypes within one single study. We analyzed the influence of the ATX-LPA axis on functional properties and IL-8, IL-6 and TNF-alpha secretion of different breast cancer cell lines categorized into five subtypes according to Dai et al.^29^: luminal A (MCF-7), luminal B (BT-474), HER2-positive (SKBR-3), triple-negative A (MDA-MB-468), and triple-negative B (MDA-MB-231). As control, non-tumorigenic epithelial mammary cells, MCF-10A, were included.

The LPA-induced cell responses varied among the investigated cell lines. Whereas MDA-MB-231, MDA-MB-468, MCF-7, and to a certain degree also SKBR-3 were stimulated dose-dependently by LPA, BT-474 and MCF-10A reacted to LPA with non-stimulated or even inhibited functional properties. The effect of LPA is mediated by signals conducted by at least six specific G protein-coupled LPAR1–6. The reason for the described different reactions of the cell lines to LPA is most likely to be found in their distinct expressions of LPARs. Multiple studies demonstrated that expression profiles vary among tumor types and subtypes^18,30^. LPAR1–3 play a crucial role in breast cancer (reviewed by^4^). Therefore, we used the LPAR1, LPAR3 and weak LPAR2 antagonist Ki16425^5,16^ to test if the above-described LPA-induced reactions were signaled mainly via LPAR1, LPAR3 (and LPAR2).

LPA stimulated significantly proliferation and migration of MDA-MB-231 cells. Further, our results showed a dose-dependent inhibition of the LPA-stimulated responses by Ki16425. Whereas previous studies found that TNBC cell lines like MDA-MB-231 express the highest levels of LPAR3 within several breast cancer subtypes ^14,15^, expression of LPAR3 by MDA-MB-231 could not be detected in other studies^18,31^. Further studies clearly demonstrated that MDA-MB-231 mainly expressed LPAR1 compared to LPAR2 and LPAR3^27,30–33^. This is in line with the RT-PCR results in the present study. MDA-MB-231 cells expressed very high levels of LPAR1, lower levels of LPAR3 and hardly LPAR2. This strongly supports the involvement of LPAR1 in LPA-induced proliferation and migration and underlines previous assumptions of a major role of LPAR1 in MDA-MB-231 cells^34^. In accordance with our migration results, dose–response studies showed that a higher dose of 1 μM LPA was associated with increased migration rates of MDA-MB-231 cells^35^. However, from a certain level of LPA concentration, a decrease in the stimulatory effect was reported, so that dose response experiments follow a bell-shaped curve^30,36^. In the present study, high concentrated LPA (1 µM) led to a higher increase in cell migration and proliferation compared to the lower concentration (0.1 µM), whereas the lower concentration of LPA evoked higher cell invasion and transmigration rates compared to the higher concentration. This corresponds with the findings of Chen et al., who observed higher invasion rates of MDA-MB-231 in 0.1 μM LPA compared to 1 μM LPA^27^. Earlier studies reported a decisive role of LPAR1 in invasion and transmigration^30,33^ and it was emphasized that efficiency in transducing signals differs between LPARs with ligand-dependent endocytosis of LPAR1 at higher concentrations of LPA^27,37^. Assuming that LPA binds primarily to LPAR1 in MDA-MB-231 cells signaling invasion and transmigration, and regarding the transmigration dose–response experiment in the present study (Supplementary Fig. S2), our observations support this theory: At 1.0 μM or higher concentrations of LPA, LPAR1 might be more rapidly endocytosed so that 1.0 μM has only a minor stimulatory effect, which cannot be sufficiently inhibited by Ki16425. Chen et al. suggested a possible cooperation of distinct LPAR. Further, efficacy of distinct LPAR seems to vary depending on the LPA concentration with LPAR2 operating predominantly at higher levels of LPA. Migration and proliferation might signal via multiple LPAR and thus, show a greater response at higher levels of LPA. Hopkins et al. have already suggested the involvement of multiple receptors in LPA-induced proliferation in MDA-MB-231 cells^34^. Additionally, similar to our results, they showed inhibition of proliferation by increasing concentrations of Ki16425.

Proliferation of MDA-MB-468 cells was significantly stimulated by LPA, while there was a lower effect on cell invasion and transmigration. In our experimental setting, the MDA-MB-468 cells expressed highest levels of LPAR3 and lower levels of LPAR2. This indicates that those receptors are also involved in signaling the induced effects. LPAR3 might thereby occupy a greater role in proliferation than in invasion and transmigration. Ki16425 mostly did not show a complete inhibition of the stimulatory effect so that our results suggest the involvement of several LPAR in mediating LPA responses. Within the investigated cell lines, MCF-7 showed a high expression level of LPAR2, which is also consistent with existing data^18,27,34^. LPA showed a stimulatory migratory effect in MCF-7 cells in the present study. Previous studies described both LPA-stimulated^28^ and LPA-inhibited invasion of MCF-7 cells^27^. Those differences might occur due to varying experimental setups, such as different incubation times or transwell coatings, or the duration of serum starvation. Whereas a significant enhancement in migration rates of MCF-7 cells was observed, there was a lesser effect on transmigration rates. Varying results of invasion/transmigration and migration might be explained by acting through different LPAR and underlying molecular pathways. In invasion/transmigration assays, LPA was added to the bottom chamber and cells in the transwell system were allowed to invade and migrate in response to a chemoattractant gradient. In contrary, the migration assay did not provide migration via chemotaxis so that differences between these assays are conceivable.

In the present study, LPAR1–3 levels of the HER2-positive SKBR-3 cells were very low and no stimulatory effect of LPA could be observed in proliferation, invasion, and transmigration assays. Previous studies described very low LPAR1 and LPAR3 expression of HER2-positive cell lines and no stimulatory effect of LPA in chemotaxis assays^18,27^. Nevertheless, the present data shows a significant LPA-induced enhancement of migration rates after 72 h in SKBR-3 cells. However, this stimulation could not be sufficiently inhibited by Ki16425. Distinct/further LPAR that are not inhibited by Ki16425 might be a reason for this observation and should be investigated in further studies. Moreover, higher concentrations of LPA might be necessary to induce a stimulatory effect. Within this study, BT-474 and MCF-10A cells reacted to LPA with non-stimulated or even inhibited transmigration and invasion comparable to previous studies^18^. Stable expressions of LPAR1 in MCF-10A cells and the acquisition of an invasive phenotype of LPA-stimulated MCF-10A cells was reported in three-dimensional experiments, nevertheless, no increasing migration rates could be observed towards LPA^31^.

ATX-LPA signaling was previously shown not only to influence cellular function, but also to activate an inflammatory cycle in breast cancer cells^19^. It was repeatedly shown that LPA crosstalks, i.a., with cytokine receptors^38^. Thereby, regulation of IL-8 and IL-6 by LPA mainly in ovarian cancer, but also in some breast cancer cell lines was described^39–42^. The upregulation of IL-8 is associated with breast cancer progression^43^ and the activation of breast stromal adipocytes^44^. Besides its tumor-promoting role including the proliferation, migration, and survival of cancer cells, IL-8 was also attributed a role in extracellular matrix remodeling, such as enhancing the immunosuppressive microenvironment and promoting epithelial-to-mesenchymal transition (EMT)^43,45,46^. IL-6 signals tumor proliferation, angiogenesis, stromal cell activation and immunomodulation of the microenvironment and might induce drug resistance^47–49^. Previous investigations reported a LPA-enhanced IL-8 production by TNBCs and MCF-7^39,41,50^, and no/slight stimulation of IL-8 production in SKBR-3 cells^40^. We therefore investigated the effect of 1.0 μM LPA on the secretion of IL-8 among the different breast (cancer) cell lines. In the present study, LPA stimulated the secretion of IL-8 in MDA-MB-468. In contrast, LPA did not enhance the secretion of IL-8 in the other investigated cell lines. LPAR2 was regarded as important receptor for signaling cytokine secretion^6^. Fang et al. suggested the LPAR2 to be most efficient in linking LPA to IL-6 and IL-8 secretion in ovarian cancer cells^40^. Moreover, Hartmann et al. proposed a LPAR2-signaled induced stimulation of the pro-inflammatory cytokines IL-6 and IL-8 in TNBC^41^. Nam et al. observed LPA-induced secretion of IL-8 in MDA-MB-231 and MDA-MB-468 cells^50^. Some of those studies used different LPA concentrations, stimulation durations and did not analyze LPAR expression of the utilized cell lines under their laboratory conditions. In the present study, MDA-MB-468 cells showed higher expression levels of LPAR2 compared e.g. to MDA-MB-231 cells. Insufficient expression of LPAR2 might have contributed to the lack of stimulation in other cell lines. However, enhanced cytokine secretion by MCF-7 cells could not be observed, indicating the involvement of further pathways or the requirement of a higher LPA stimulation dose or a different incubation period.

TNF-alpha plays a dual role in cancer and can inhibit and enhance tumor progression. It was shown to induce apoptosis and necrosis of malignant cells and to facilitate drug accumulation at the tumor site^51^. On the other hand, TNF-alpha is an important pro-inflammatory cytokine in the tumor microenvironment and is secreted both by breast cancer and by surrounding stromal cells. It is attributed a role in tumor proliferation, tumorangiogenesis and EMT in breast cancer^51^. Further, TNF-alpha enhances ATX production in the adjacent adipose tissue^19^. A recent study showed that LPA induces secretion of TNF-alpha in ovarian cancer cells and thus, adjusts an inflammatory network in ovarian cancer^52^. However, to our knowledge, no prior study has revealed an LPA-induced TNF-alpha secretion in breast cancer cells. Whereas we observed an enhanced release of TNF-alpha in MDA-MB-468 cells, we found a decreased secretion of TNF-alpha in MDA-MB-231 cells after incubation with LPA. Wang et al. established the involvement of LPAR2 in the mediation of TNF-alpha induction and reported a minor or even inhibitory role of LPAR3 in TNF-alpha release in ovarian cancer cell lines^52^. LPA-induced secretion of TNF-alpha in MDA-MB-468 and the corresponding LPAR expression profile of this cell line in the present study support the proposed involvement of LPAR2 in TNF-alpha production. Since LPAR3 is most highly expressed in MDA-MB-468, the reported potential inhibitory effect of LPAR3 does not appear to be strong enough to suppress LPAR2-mediated stimulation completely. The inhibition of TNF-alpha in MDA-MB-231 cells might indicate an inhibitory function of LPAR1. However, further and more detailed cytokine analyses are necessary to elucidate the underlying pathways.

The described differences in LPA-mediated cellular responses and IL-8, IL-6 and TNF-alpha expression among the breast (cancer) cells underline previous findings that ATX-LPA-LPAR signaling is a complex issue, as it depends on the interplay on various factors, such as expression of LPAR, LPA concentration, and the same LPAR showing tumor-promoting as well as anti-tumorigenic effects. Several underlying downstream signaling pathways have already been identified^53,54^. Nevertheless, further research including further LPAR inhibitors and providing mechanistic insights into underlying molecular pathways is needed to fully elucidate the complex interactions. Additionally, the role of LPA turnover by lipid phosphate phophatases (LPPs) in different cell lines and the role of the tumor microenvironment should not be unattended in this issue and be included in further investigations.

## Conclusions

Among the examined breast cancer subtypes, LPA dose-dependently stimulated tumor-promoting cellular functions of triple-negative A, triple-negative B, luminal A, and to a certain degree also HER-2 positive subtypes, whereas the luminal B subtype and the non-tumorigenic epithelial cell line were not stimulated by LPA. Depending on the functional assay, Ki16425 inhibited the LPA-induced stimulation in triple-negative breast cancer and luminal A cell lines in a variable intensity and enhanced secretion of IL-8, IL-6 and TNF-alpha in MDA-MB-468 cells. This indicates the interplay of different LPAR in the performed assays. The present study further elucidates the role of the ATX-LPA axis in breast cancer and might contribute to identify suitable subtypes for a promising targeted therapy.

## Supplementary Information

Supplementary Legends.

Supplementary Figure S1.

Supplementary Figure S2.

## Data Availability

The datasets generated during and/or analyzed during the current study are available from the corresponding author on reasonable request.

## References

[1] Tokumura A (2002). Identification of human plasma lysophospholipase D, a lysophosphatidic acid-producing enzyme, as autotaxin, a multifunctional phosphodiesterase. J. Biol. Chem..

[2] Akira T, Kengo H, Kenji F, Hiroaki T (1986). Involvement of lysophospholipase D in the production of lysophosphatidic acid in rat plasma. Biochim. Biophys. Acta (BBA) Lipids Lipid Metab..

[3] Stefan C, Jansen S, Bollen M (2005). NPP-type ectophosphodiesterases: Unity in diversity. Trends Biochem. Sci..

[4] Xiang H, Lu Y, Shao M, Wu T (2020). Lysophosphatidic acid receptors: Biochemical and clinical implications in different diseases. J. Cancer.

[5] Yung YC, Stoddard NC, Chun J (2014). LPA receptor signaling: Pharmacology, physiology, and pathophysiology. J. Lipid Res..

[6] Panupinthu N, Lee HY, Mills GB (2010). Lysophosphatidic acid production and action: Critical new players in breast cancer initiation and progression. Br. J. Cancer.

[7] Samadi N, Gaetano C, Goping IS, Brindley DN (2009). Autotaxin protects MCF-7 breast cancer and MDA-MB-435 melanoma cells against taxol-induced apoptosis. Oncogene.

[8] Samadi N, Bekele RT, Goping IS, Schang LM, Brindley DN (2011). Lysophosphatidate induces chemo-resistance by releasing breast cancer cells from taxol-induced mitotic arrest. PLoS ONE.

[9] Brindley DN, Lin FT, Tigyi GJ (1831). Role of the autotaxin-lysophosphatidate axis in cancer resistance to chemotherapy and radiotherapy. Biochim. Biophys. Acta.

[10] Brindley DN, Tang X, Meng G, Benesch MGK (2020). Role of adipose tissue-derived autotaxin, lysophosphatidate signaling, and inflammation in the progression and treatment of breast cancer. Int. J. Mol. Sci..

[11] Boucharaba A (2009). Bioactive lipids lysophosphatidic acid and sphingosine 1-phosphate mediate breast cancer cell biological functions through distinct mechanisms. Oncol. Res..

[12] Liu S (2009). Expression of autotaxin and lysophosphatidic acid receptors increases mammary tumorigenesis, invasion, and metastases. Cancer Cell.

[13] Wang J (2016). Roles of LPA receptor signaling in breast cancer. Expert Rev. Mol. Diagn..

[14] Sun K (2015). Aberrant expression and potential therapeutic target of lysophosphatidic acid receptor 3 in triple-negative breast cancers. Clin. Exp. Med..

[15] Popnikolov NK, Dalwadi BH, Thomas JD, Johannes GJ, Imagawa WT (2012). Association of autotaxin and lysophosphatidic acid receptor 3 with aggressiveness of human breast carcinoma. Tumour Biol..

[16] Ohta H (2003). Ki16425, a subtype-selective antagonist for EDG-family lysophosphatidic acid receptors. Mol. Pharmacol..

[17] David M (2012). Targeting lysophosphatidic acid receptor type 1 with Debio 0719 inhibits spontaneous metastasis dissemination of breast cancer cells independently of cell proliferation and angiogenesis. Int. J. Oncol..

[18] Schmid R (2018). ADSCs and adipocytes are the main producers in the autotaxin-lysophosphatidic acid axis of breast cancer and healthy mammary tissue in vitro. BMC Cancer.

[19] Benesch MG (2015). Tumor-induced inflammation in mammary adipose tissue stimulates a vicious cycle of autotaxin expression and breast cancer progression. Faseb J.

[20] Benesch MGK, Tang X, Brindley DN (2020). Autotaxin and breast cancer: Towards overcoming treatment barriers and sequelae. Cancers (Basel)..

[21] Kengelbach-Weigand A (2019). Plasticity of patient-matched normal mammary epithelial cells is dependent on autologous adipose-derived stem cells. Sci. Rep..

[22] Schindelin J (2012). Fiji: An open-source platform for biological-image analysis. Nat. Methods.

[23] Tilli TM, Castro Cda S, Tuszynski JA, Carels N (2016). A strategy to identify housekeeping genes suitable for analysis in breast cancer diseases. BMC Genomics.

[24] Benesch MG (2015). Autotaxin is an inflammatory mediator and therapeutic target in thyroid cancer. Endocr. Relat. Cancer.

[25] Erstad DJ, Tager AM, Hoshida Y, Fuchs BC (2017). The autotaxin-lysophosphatidic acid pathway emerges as a therapeutic target to prevent liver cancer. Mol. Cell Oncol..

[26] Tanyi JL (2003). The human lipid phosphate phosphatase-3 decreases the growth, survival, and tumorigenesis of ovarian cancer cells: Validation of the lysophosphatidic acid signaling cascade as a target for therapy in ovarian cancer. Cancer Res..

[27] Chen M, Towers LN, O'Connor KL (2007). LPA2 (EDG4) mediates Rho-dependent chemotaxis with lower efficacy than LPA1 (EDG2) in breast carcinoma cells. Am. J. Physiol. Cell Physiol..

[28] Sun K (2016). Curcumin inhibits LPA-induced invasion by attenuating RhoA/ROCK/MMPs pathway in MCF7 breast cancer cells. Clin. Exp. Med..

[29] Dai X, Cheng H, Bai Z, Li J (2017). Breast cancer cell line classification and its relevance with breast tumor subtyping. J. Cancer.

[30] Hama K (2004). Lysophosphatidic acid and autotaxin stimulate cell motility of neoplastic and non-neoplastic cells through LPA_1_. J. Biol. Chem..

[31] Li TT (2009). Beta-arrestin/Ral signaling regulates lysophosphatidic acid-mediated migration and invasion of human breast tumor cells. Mol. Cancer Res..

[32] Sahay D (2015). The LPA1/ZEB1/miR-21-activation pathway regulates metastasis in basal breast cancer. Oncotarget.

[33] Kim JH, Adelstein RS (2011). LPA(1) -induced migration requires nonmuscle myosin II light chain phosphorylation in breast cancer cells. J. Cell Physiol..

[34] Hopkins MM, Zhang Z, Liu Z, Meier KE (2016). Eicosopentaneoic acid and other free fatty acid receptor agonists inhibit lysophosphatidic acid- and epidermal growth factor-induced proliferation of human breast cancer cells. J. Clin. Med..

[35] Du J (2010). Lysophosphatidic acid induces MDA-MB-231 breast cancer cells migration through activation of PI3K/PAK1/ERK signaling. PLoS ONE.

[36] Shida D (2003). Lysophosphatidic acid (LPA) enhances the metastatic potential of human colon carcinoma DLD1 cells through LPA1. Cancer Res..

[37] Murph MM, Scaccia LA, Volpicelli LA, Radhakrishna H (2003). Agonist-induced endocytosis of lysophosphatidic acid-coupled LPA1/EDG-2 receptors via a dynamin2- and Rab5-dependent pathway. J. Cell Sci..

[38] Xu Y (2019). Targeting lysophosphatidic acid in cancer: The issues in moving from bench to bedside. Cancers.

[39] Schwartz BM (2001). Lysophospholipids increase interleukin-8 expression in ovarian cancer cells. Gynecol. Oncol..

[40] Fang X (2004). Mechanisms for lysophosphatidic acid-induced cytokine production in ovarian cancer cells. J. Biol. Chem..

[41] Hartman ZC (2013). Growth of triple-negative breast cancer cells relies upon coordinate autocrine expression of the proinflammatory cytokines IL-6 and IL-8. Cancer Res..

[42] Chou CH (2005). Up-regulation of interleukin-6 in human ovarian cancer cell via a Gi/PI3K-Akt/NF-kappaB pathway by lysophosphatidic acid, an ovarian cancer-activating factor. Carcinogenesis.

[43] Todorović-Raković N, Milovanović J (2013). Interleukin-8 in breast cancer progression. J. Interferon Cytokine Res..

[44] Al-Khalaf HH (2019). Interleukin-8 activates breast cancer-associated adipocytes and promotes their angiogenesis- and tumorigenesis-promoting effects. Mol. Cell Biol..

[45] Han Z-J (2021). Roles of the CXCL8-CXCR1/2 axis in the tumor microenvironment and immunotherapy. Molecules.

[46] David JM, Dominguez C, Hamilton DH, Palena C (2016). The IL-8/IL-8R axis: A double agent in tumor immune resistance. Vaccines (Basel).

[47] Taher MY, Davies DM, Maher J (2018). The role of the interleukin (IL)-6/IL-6 receptor axis in cancer. Biochem. Soc. Trans..

[48] Ghandadi M, Sahebkar A (2016). Interleukin-6: A critical cytokine in cancer multidrug resistance. Curr. Pharm. Des..

[49] Masjedi A (2018). The significant role of interleukin-6 and its signaling pathway in the immunopathogenesis and treatment of breast cancer. Biomed. Pharmacother..

[50] Nam JS (2018). Lysophosphatidic acid enhances breast cancer cells-mediated osteoclastogenesis. Korean J. Physiol. Pharmacol..

[51] Cruceriu D, Baldasici O, Balacescu O, Berindan-Neagoe I (2020). The dual role of tumor necrosis factor-alpha (TNF-α) in breast cancer: Molecular insights and therapeutic approaches. Cell. Oncol..

[52] Wang W (2020). Lysophosphatidic acid induces tumor necrosis factor-alpha to regulate a pro-inflammatory cytokine network in ovarian cancer. FASEB J..

[53] Lin Y-H, Lin Y-C, Chen C-C (2021). Lysophosphatidic acid receptor antagonists and cancer: The current trends, clinical implications, and trials. Cells.

[54] Aiello S, Casiraghi F (2021). Lysophosphatidic acid: Promoter of cancer progression and of tumor microenvironment development. A promising target for anticancer therapies?. Cells.

