# Supplementary material for: Influence of the autotaxin-lysophosphatidic acid axis on cellular function and cytokine expression in different breast cancer cell lines
Source: Sci Rep. 2022 Apr 1;12:5565. doi: 10.1038/s41598-022-09565-3 (PMC8975816; doi:10.1038/s41598-022-09565-3)
Supplement: Supplementary file 1 — Supplementary Legends. [file 41598_2022_9565_MOESM1_ESM.docx]

**Supplementary Fig. S1:** Ki16425 control groups

Ki16425 controls including 2 µM and 20 µM Ki16425 for all cell lines and all assays.

**Supplementary Fig. S2:** Dose response analysis

Effect of increasing concentrations of lysophosphatidic acid (LPA) 18:1 on transmigration of MDA-MB-231 cells (control=1). Columns show the ratio of transmigrated cells compared to the control group (y-axis) in varying concentrations of LPA (x-axis). n=2 replicate experiments. Values are presented as mean and their SD.
