# Supplementary figures and images for: Influence of the autotaxin-lysophosphatidic acid axis on cellular function and cytokine expression in different breast cancer cell lines
Source: Sci Rep. 2022 Apr 1;12:5565. doi: 10.1038/s41598-022-09565-3 (PMC8975816; doi:10.1038/s41598-022-09565-3)

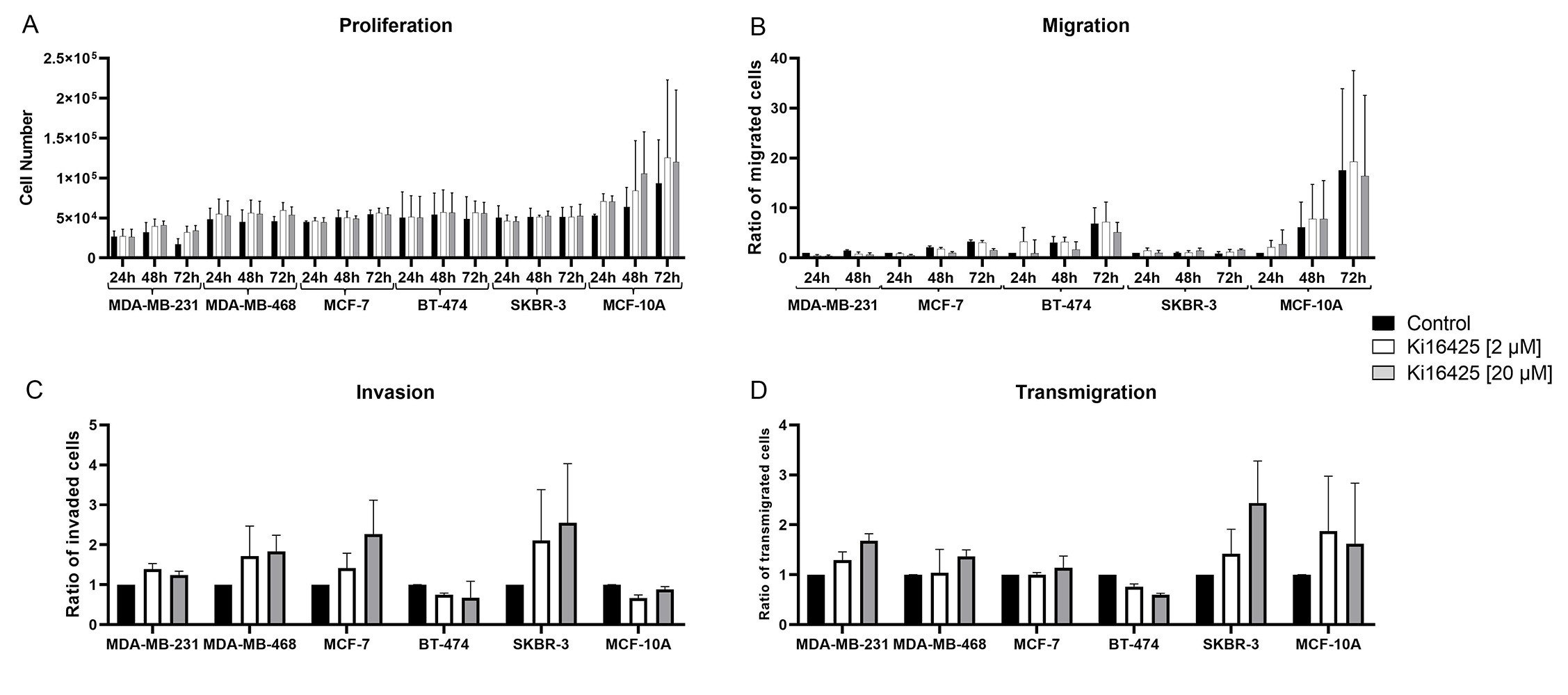

Supplement: Supplementary file 2 — Supplementary Figure S1. [file 41598_2022_9565_MOESM2_ESM.tif]

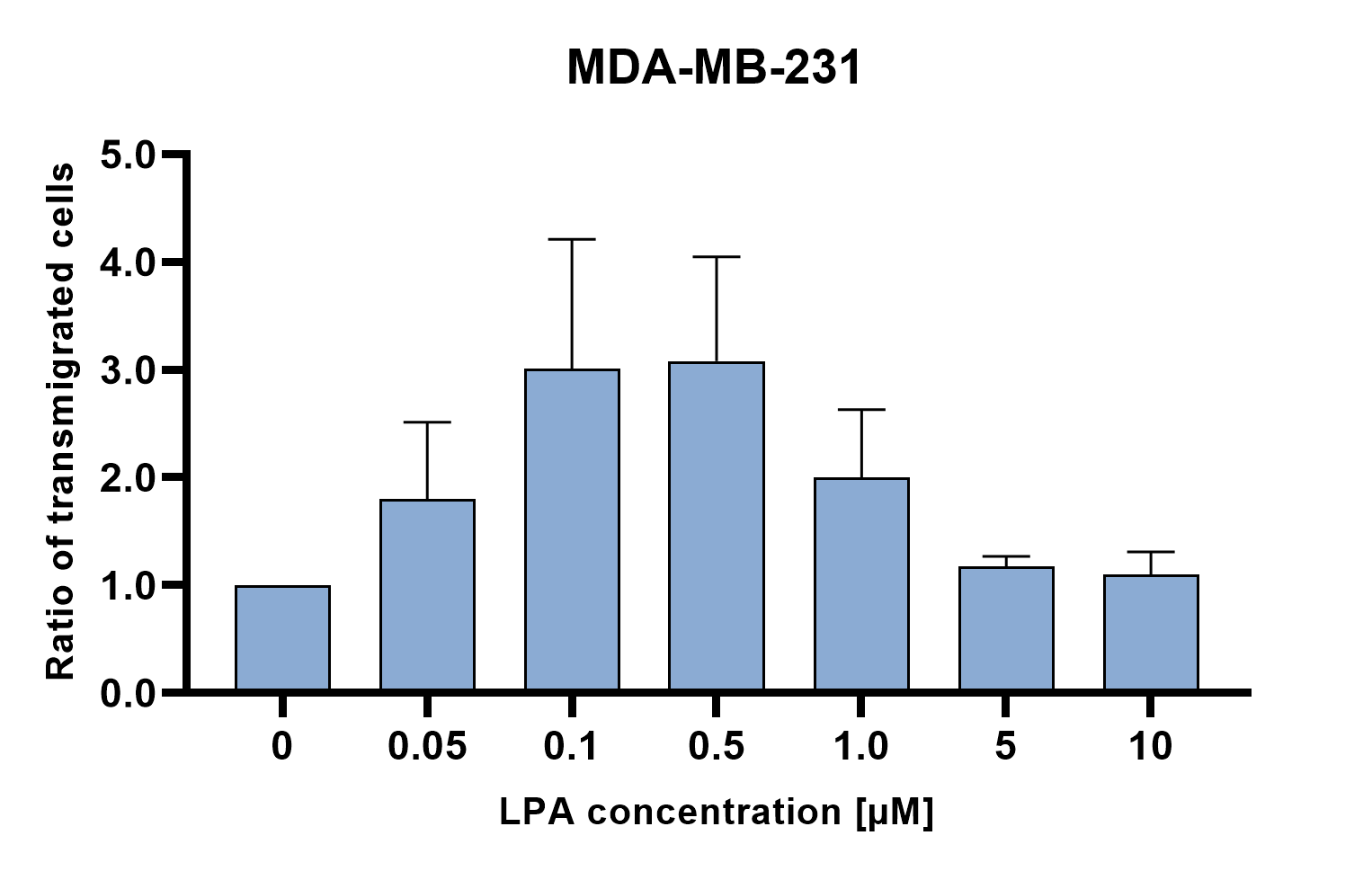

Supplement: Supplementary file 3 — Supplementary Figure S2. [file 41598_2022_9565_MOESM3_ESM.tif]
